# Fentanyl Test Strip Use and Overdose Risk Reduction Behaviors Among People Who Use Drugs

**DOI:** 10.1001/jamanetworkopen.2025.10077

**Published:** 2025-05-13

**Authors:** Rachel A. Vickers-Smith, Kitty H. Gelberg, Janet E. Childerhose, Denise C. Babineau, Redonna Chandler, James L. David, Lauren D’Costa, Megan Dzurec, Barry Eggleston, Amanda Fallin-Bennett, Laura C. Fanucchi, Soledad Fernandez, Jace Gilbert, Louisa Gilbert, Megan E. Hall, Brooke E. Hiltz, Michael W. Konstan, Kathryn E. Lancaster, Beth Linas, Katherine R. Marks, Nichole Michaels, Jennifer Miles, Fernando Montero, Haley J. Ramsey Harden, Carter Roeber, Mary R. Russo, Rachel Taylor, Melissa A. Theis, Jennifer Villani, Emmanuel Oga, Nabila El-Bassel, Sharon L. Walsh, Bridget Freisthler

**Affiliations:** 1 College of Public Health, University of Kentucky, Lexington; 2 School of Social Work, Columbia University, New York, New York; 3 Division of Internal Medicine, The Ohio State University College of Medicine, Columbus; 4 Research Triangle Institute International, Research Triangle Park, North Carolina; 5 National Institute on Drug Abuse, Bethesda, Maryland; 6 The Ohio State University College of Medicine, Columbus; 7 College of Nursing, University of Kentucky, Lexington; 8 Department of Internal Medicine, University of Kentucky College of Medicine, Center on Drug and Alcohol Research, University of Kentucky, Lexington; 9 Biomedical Informatics and Center for Biostatistics, The Ohio State University, Columbus; 10 Substance Use Priority Research Area, University of Kentucky, Lexington,; 11 School of Medicine, Case Western Reserve University, Cleveland, Ohio; 12 Department of Implementation Science, Wake Forest University School of Medicine, Winston-Salem, North Carolina; 13 Kentucky Department for Behavioral Health, Developmental and Intellectual Disabilities, Frankfort; 14 Center for Injury Research and Policy, Abigail Wexner Research Institute at Nationwide Children's Hospital, Columbus; 15 Substance Use Priority Research Area, University of Kentucky, Lexington; 16 Social Intervention Group, Columbia University, New York, New York; 17 Substance Use Priority Research Area, University of Kentucky, Lexington; 18 Substance Abuse and Mental Health Services Administration, Rockville, Maryland; 19 Now with Federal Bureau of Prisons, Lexington, Kentucky; 20 Now with The Geneva Foundation, Lackland Air Force Base, Texas; 21 Department of Behavioral Science, Center on Drug and Alcohol Research, University of Kentucky College of Medicine, University of Kentucky, Lexington; 22 Now with College of Social Work, University of Tennessee, Knoxville

## Abstract

**Importance:**

Illegal fentanyl is driving overdose mortality, and fentanyl test strips (FTS) can be used to test drugs for fentanyl at the point of consumption. Evidence on whether FTS use is associated with overdose risk reduction behaviors is encouraging, but largely limited to smaller, single-site studies.

**Objective:**

To determine whether self-reported baseline FTS use among people who use drugs (PWUD) was associated with overdose risk reduction behaviors and nonfatal overdose over a 28-day follow-up.

**Design, Setting, and Participants:**

Multisite, observational cohort study of PWUD conducted from May to December 2023 as an ancillary study of the HEALing Communities Study, which consists of fixed and mobile direct service provision sites in 14 community partner organizations distributing FTS. Participants lived in Kentucky, New York, or Ohio and reported using heroin, fentanyl, cocaine, methamphetamine, or nonprescribed opioids, benzodiazepines, or stimulants within 30 days before baseline. Participants were followed up for a maximum of 37 days.

**Exposure:**

Baseline FTS use.

**Main Outcome and Measures:**

The primary outcome was a composite score measuring the self-reported number and frequency of using 8 overdose risk reduction behaviors. Secondary outcomes included multiple measures (eg, self-reported nonfatal overdose).

**Results:**

The study included 732 participants (median [IQR] age, 41 [34.0-48.0] years; 369 [50.4%] male; 64 [8.9%] Black or African American, 587 [81.3%] White, and 71 [9.8%] other races); 414 reported baseline FTS use and 318 did not. Compared with nonusers, a higher percentage of baseline FTS users were from Ohio and White, while a lower percentage were from New York and Hispanic and/or Black. In adjusted analyses, PWUD who used FTS had a mean daily composite score for overdose risk reduction behaviors that was 0.86 (95% CI, 0.34-1.38) units higher across follow-up compared with nonusers (score for FTS users, 7.37; nonusers, 6.51). There was no difference in self-reported nonfatal overdoses between the 2 groups (mean daily risk for FTS users, 0.02; nonusers, 0.02; risk ratio, 1.20; 95% CI, 0.70-2.06).

**Conclusions and Relevance:**

In this cohort study, baseline FTS use was associated with greater engagement in overdose risk reduction behaviors during follow-up, but not with the risk of nonfatal overdose during follow-up, suggesting PWUD who use FTS may also engage in a broader set of harm reduction strategies.

## Key Points

**Question:**

Is fentanyl test strip (FTS) use associated with engaging in overdose risk reduction behaviors among people who use drugs (PWUD)?

**Findings:**

In this multisite cohort study of 732 PWUD, participants who reported baseline FTS use were significantly more likely to report more overdose risk reduction behaviors more frequently compared with participants who reported not using FTS at baseline.

**Meaning:**

These findings suggest that FTS use is associated with overdose risk reduction behaviors, demonstrating the role of FTS as a harm reduction strategy among PWUD.

## Teaser

This cohort study evaluates whether self-reported baseline fentanyl test strip use among people who use drugs was associated with overdose risk reduction behaviors and nonfatal overdose over a 28-day follow-up.

## Introduction

The US opioid overdose crisis has reached unprecedented levels, largely driven by the proliferation of fentanyl in the street drug supply.^1^ Fentanyl, a synthetic opioid 50 times more potent than heroin, is often mixed with other drugs, increasing overdose risk among people who use drugs (PWUD). Since 2013, fentanyl has become a pervasive adulterant of the illicit drug supply,^2^ identified in heroin, methamphetamine, cocaine, counterfeit prescription pills (eg, opioids and benzodiazepines), and others.^3,4^ Recent national forensic data indicate that fentanyl is present in 40% to 50% of heroin samples, and while less common in nonopioid substances, its presence has risen regionally, exceeding 10% in cocaine and 20% in methamphetamine in some areas.^5^ In the US, fentanyl is commonly injected or smoked, with smoking becoming increasingly prevalent in some regions.^6,7^

In response to fentanyl’s widespread presence, fentanyl test strips (FTS) have been introduced as a harm reduction strategy, allowing individuals to test their drugs for the presence of fentanyl and potentially mitigate overdose risk.^8,9,10^ FTS are rapid immunoassays designed to test urine, but have been repurposed to test individual drug samples for fentanyl.^8,9,10^ Using a small amount of drug residue and water, PWUD can use these paper strips to detect fentanyl concentrations of approximately 20 to 200 mg/mL in less than 5 minutes. FTS have high sensitivity in detecting fentanyl and fentanyl analogues in street drugs, although stimulants and cutting agents can sometimes interfere with results.^11,12,13^

Research documents high levels of acceptability and willingness to use FTS among PWUD, particularly those who express fear of fentanyl or overdose.^14^ Furthermore, FTS use is associated with behaviors that reduce overdose risk, like using smaller amounts of fentanyl-positive drugs, testing drugs before use, using when others are present, and ensuring naloxone is available.^14^ Although evidence on FTS as an acceptable harm reduction tool is encouraging, it is also limited. Specifically, there is a lack of robust data on the actual uptake of FTS and their association with harm reduction behaviors and overdose occurrence, as many existing studies were qualitative or cross-sectional, had relatively small sample sizes, and/or were restricted to a single geographical area.^14^ These limitations underscore the need for large-scale observational studies to better understand FTS use in general population settings and with diverse populations.

To address these gaps, we conducted a prospective, multisite, observational study to examine the association between baseline FTS use and engagement in overdose risk reduction behaviors, as well as self-reported nonfatal overdoses, among a cohort of PWUD in 3 states. We hypothesized that those who used FTS at baseline would engage in a greater quantity and frequency of overdose risk reduction behaviors during follow-up compared with those who did not use FTS. We also expected the mean daily risk of self-reported nonfatal overdose would be lower during follow-up among participants who used FTS at baseline.

## Methods

### Study Design

The Stay Safe Study is an ancillary study of the Healing Communities Study (HCS).^15,16^ The Stay Safe Study received ethics approval from Advarra’s institutional review board. The Strengthening the Reporting of Observational Studies in Epidemiology (STROBE) reporting guidelines were followed. The study period was August 2022 to June 2024 with recruitment and data collection occurring May to December 2023.

After providing written informed consent via an electronic tablet, participants completed a baseline survey and completed 4 weekly surveys over a 28-day observation period (referred to as follow-up). The weekly surveys used a modified timeline followback approach^17,18^ to capture self-reported patterns of FTS use, drug use, overdose risk reduction behaviors, and nonfatal overdose events on each day they used drugs in the past week. Participants were compensated up to $250 for completing the screening, baseline, and approximately 30-minute weekly surveys. Compensation was provided after each activity, and participants received payment for surveys they started, even if they did not complete them.

### Setting

Fourteen community partner organizations that distribute FTS in 3 states participated and hosted data collection activities at their sites (5 in Kentucky, 3 in New York, and 6 in Ohio). These included a range of fixed and mobile locations that deliver harm reduction services.

### Participants

Eligibility criteria included being aged 18 years or older, self-assessed ability to read and speak English proficiently, ability to understand and provide consent, current residence in and plan to continue residing in Kentucky, New York, or Ohio during follow-up, and report past 30-day drug use (see Variables and Measurement for drug list). The Figure summarizes those screened, eligible, enrolled, and followed up during the study. To be considered enrolled, participants were required to complete the approximately 30-minute baseline survey; completion of a survey was defined as answering at least 1 question. To be included in the analytic sample (referred to as the full sample), participants had to have self-reported drug use on 1 or more days and have completed 2 or more weekly surveys during follow-up.

**Figure.**
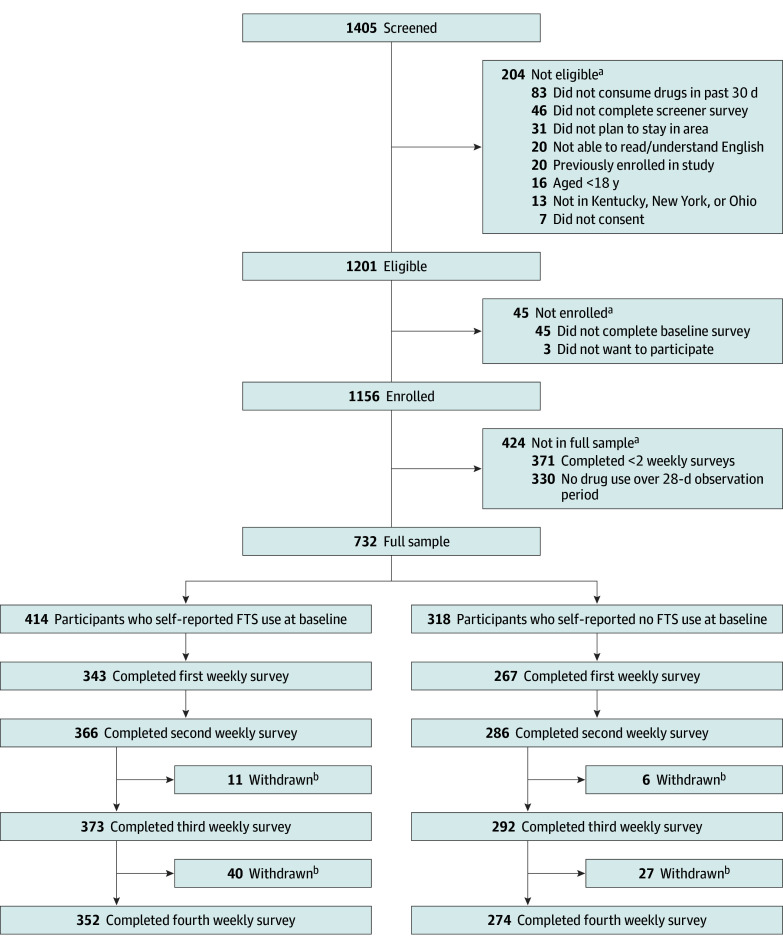
Flow Diagram of Those Screened, Eligible, Enrolled, and Followed During the Study ^a^Reasons for exclusion from sample are not mutually exclusive. ^b^Participants withdrawn from the study were lost to follow-up. FTS indicates fentanyl test strip.

### Variables and Measurement

#### Outcomes

##### Overdose Risk Reduction Behaviors

The primary outcome was a composite score measuring the number and frequency of 8 overdose risk reduction behaviors on each day drugs were used (Table 1).^19,20,21,22,23,24,25,27,28,29,30,31,32,33,34^ These behaviors were (1) commonly recommended in the literature to reduce overdose risk, and (2) independent from one another. Participants indicated whether they always, sometimes, or never engaged in each behavior that day, and scored accordingly (Table 1). The daily composite score summed frequencies across all 8 overdose risk reduction behaviors, ranging from 0 (ie, did not engage in any behaviors that day) to 15 (ie, engaged in all behaviors every time drugs were used that day). One behavior, “threw out the drugs because they were bad or not what you expected,” could only contribute 0 or 1 to the score because if a participant always discarded drugs, then they would not have used any drugs that day (Table 1).

**Table 1.** Description and References for the Daily Overdose Risk Reduction Behaviors Included in Calculating the Primary Outcome Composite Score^a^

Overdose risk reduction behavior	References
Someone else was present when you used drugs	Urmanche et al,^19^ 2022; Goldman et al,^20^ 2019; Klaire et al,^21^ 2022; Mema et al,^22^ 2018; and Mistler et al,^23^ 2021
Naloxone was nearby when you used drugs	Goldman et al,^20^ 2019; Mema et al,^22^ 2018; McKnight et al,^24^ 2018; and Frost et al,^25^ 2022
Test the strength of your drugs before using them^b^	Peiper et al,^9^ 2019; Park et al,^26^ 2021; Urmanche et al,^19^ 2022; Walters et al,^27^ 2023; and Park et al,^28^ 2020
Use your drugs slowly^c^	Peiper et al,^9^ 2019; Park et al,^26^ 2021; Klaire et al,^21^ 2022; Mema et al,^22^ 2018; Mistler et al,^23^ 2021; Frost et al^25^ 2022; and Park et al,^28^ 2020
Ask someone to check on you when using drugs	Park et al,^26^ 2021; Urmanche et al,^19^ 2022; and Park et al,^28^ 2020
Smoke, snort, sniff, swallow, or anally use your drugs	Peiper et al,^9^ 2019 and Walters et al,^27^ 2023
Watch someone use the same drugs to see how they reacted before you used them	Stein et al,^29^ 2019 and Mars et al,^30^ 2018
Throw out drugs because they were bad or not what you expected	Goldman et al,^20^ 2019; Mema et al,^22^ 2018; Mistler et al,^23^ 2021; Frost et al,^25^ 2022; Park et al,^28^ 2020; Goodman-Meza et al,^31^ 2022; Reed et al,^32^ 2022; Weicker et al,^33^ 2020; and Oh et al,^34^ 2020

^a^ Scoring was always (2 points), sometimes (1 point), and never (0 points) for all items except "Throw out drugs because they were bad or not what you expected," which only included scores for sometimes (1 point) and never (0 points).

^b^ Testing the strength of drugs may involve using a small amount first to gauge its effects before proceeding (eg, taking a small dose orally, administering a partial injection or test shot, or insufflating a small amount).

^c^ Using your drugs slowly involves deliberately spacing out use over time (eg, taking smaller doses gradually) to monitor effects.

On each drug use day, we analyzed the following secondary outcomes: (1) the overall number and (2) each individual behavior reported. For these analyses, responses of “always” or “sometimes” collapsed into a single yes category, contributing to the behavior count for that day.

##### Nonfatal Overdose

This was self-reported on the 4 weekly surveys. On each drug use day, participants were prompted to report a time in the past week “when they lost consciousness, and someone had to do something to revive them.”

#### Exposure

##### Baseline FTS Use

The primary exposure was self-reported past 30-day FTS use (yes or no) measured at baseline. Individuals who did not respond, or indicated “don’t know” or “prefer not to answer” were categorized as not using FTS.

#### Covariates

Covariates included in the model for each outcome were prespecified before analysis based on study design and expert opinion. All response options for covariates are described in detail in the eMethods in Supplement 1.

##### Sociodemographic and Drug Use Characteristics

Sociodemographic characteristics were self-reported on the baseline survey, including state of residence, age, self-reported race (Black or African American, White, or Other [American Indian or Alaska Native, Asian, Native Hawaiian or Other Pacific Islander, or some other race]), self-reported ethnicity (Hispanic or Latino or not Hispanic or Latino), sex, highest level of education completed, employment status, ever received FTS instructions, and past 30-day overdose. Given that overdose rates are declining overall but increasing among certain racial and ethnic groups,^35^ we included race and ethnicity in our analyses to account for differential risk due to unmeasured factors such as unequal access to care and structural barriers. We collected both sex at birth and gender identity at baseline; we chose to include only sex in analyses because of the differential overdose risk likely conferred by biological sex^36^ and high concordance with gender in our study.

##### Characteristics Varying Over Time

Characteristics were self-reported on a daily basis in the weekly surveys and included study week, part of week, number of times FTS were used, injection drug use, and drug category used. Questions on drug use included heroin, fentanyl, powder cocaine, crack or rock cocaine, methamphetamine, pain pills or opioids not prescribed for participant, benzodiazepines not prescribed for participant, and stimulants not prescribed for participant. Drug category used was then defined by collapsing responses to these questions into 5 mutually exclusive categories: illicit opioids only; illicit stimulants only; illicit opioids and illicit stimulants only; illicit benzodiazepines; or no drug use/do not know/prefer not to answer (see eMethods in Supplement 1 for further description).

### Statistical Analysis

We calculated descriptive statistics for baseline characteristics and compared between baseline FTS users vs nonusers using a Pearson χ^2^ test or Fisher exact test for categorical variables and a Wilcoxon rank sum test for continuous variables. Population-averaged estimates with 95% CIs were assessed using generalized estimating equations (GEE) with appropriate distributions and link functions. Models included baseline FTS use as the primary exposure along with prespecified baseline and time-varying covariates (see eMethods in Supplement 1 for a full listing of covariates). The covariance structure for residual effects was selected based on model fit criteria. Interaction terms were tested to assess association modification. A generalized linear mixed model that assumes data are missing at random was fit to the composite score to assess potential bias arising from missing data, with negligible differences. All hypotheses were tested using a 2-sided significance level of .05. Consequently, all *P* values presented are purely descriptive. All analyses were conducted using SAS Version 9.4 (SAS Institute) and R Version 4.3.3 (R Project for Statistical Computing).

## Results

Of the 1156 participants enrolled, 732 self-reported drug use on 1 or more days and completed 2 or more weekly surveys during follow-up and thus were included in the full sample (Figure). Survey completion rates are shown in eTable 1 in Supplement 1. Baseline characteristics were compared among enrolled participants between those included and excluded from the full sample (eTable 2 in Supplement 1).

### Baseline Characteristics

The study included 732 participants (median [IQR] age, 41 [34.0-48.0] years; 369 [50.4%] male; 64 [8.9%] Black or African American, 587 [81.3%] White, and 71 [9.8%] other races). Over half the sample (414 participants [56.6%]) reported baseline FTS use, and 318 did not (Table 2). Compared with nonusers, a higher percentage of baseline FTS users were from Ohio and White, while a lower percentage were from New York and Hispanic and/or Black (Table 2). Baseline FTS users also had a higher relative frequency of past 30-day fentanyl, heroin, methamphetamine, and opioid-stimulant combination use, and a greater proportion of them engaged in injection drug use (Table 2).

**Table 2.** Baseline Characteristics of the Full Sample by Self-Reported Baseline Fentanyl Test Strip (FTS) Use

Characteristic	Participants, No. (%)			
	Full sample (N = 732)	FTS use at baseline (n = 414)	No FTS use at baseline (n = 318)	*P* value
Sociodemographic characteristics				
State				
Kentucky	254 (34.7)	138 (33.3)	116 (36.5)	.001
New York	162 (22.1)	75 (18.1)	87 (27.4)	
Ohio	316 (43.2)	201 (48.6)	115 (36.2)	
Age				
Total respondents, No.^a^	732	414	318	.68
Median (IQR)	41 (34.0-48.0)	41 (34.0-48.0)	40 (33.0-49.0)	
Race				
Total respondents, No.^a^	722	409	313	.001
Black or African American	64 (8.9)	25 (6.1)	39 (12.5)	
White	587 (81.3)	351 (85.8)	236 (75.4)	
Other^b^	71 (9.8)	33 (8.1)	38 (12.1)	
Ethnicity				
Total respondents, No.^a^	724	409	315	.001
Hispanic or Latino	64 (8.8)	23 (5.6)	41 (13.0)	
Not Hispanic or Latino	660 (91.2)	386 (94.4)	274 (87.0)	
Sex assigned at birth				
Total respondents, No.^a^	732	414	318	.98
Female	363 (49.6)	206 (49.8)	157 (49.4)	
Male	369 (50.4)	208 (50.2)	161 (50.6)	
Highest level of education completed				
Total respondents, No.^a^	725	407	318	.29
Less than high school	73 (10.1)	42 (10.3)	31 (9.7)	
Some high school, high school graduate, or GED	417 (57.5)	224 (55.0)	193 (60.7)	
Some college or higher	235 (32.4)	141 (34.6)	94 (29.6)	
Employment status				
Total respondents, No.^a^	715	406	309	.25
Unemployed	391 (54.7)	218 (53.7)	173 (56.0)	
Employed part- or full-time	169 (23.6)	105 (25.9)	64 (20.7)	
Other (eg, retired, disabled, student, or homemaker)	155 (21.7)	83 (20.4)	72 (23.3)	
Past 30-d drug use^a^^,^^c^				
Fentanyl (n = 698)	489 (70.1)	312 (78.2)	177 (59.2)	<.001
Heroin (n = 727)	511 (70.3)	317 (76.8)	194 (61.8)	<.001
Powder cocaine (n = 725)	294 (40.6)	164 (39.9)	130 (41.4)	.74
Crack or rock cocaine (n = 727)	368 (50.6)	209 (51.0)	159 (50.2)	.89
Methamphetamine (n = 725)	442 (61.0)	265 (64.5)	177 (56.4)	.03
Unprescribed opioids (n = 726)	229 (31.5)	136 (33.1)	93 (29.5)	.35
Unprescribed benzodiazepines (n = 721)	197 (27.3)	119 (29.2)	78 (24.9)	.24
Unprescribed stimulants (n = 729)	93 (12.8)	56 (13.6)	37 (11.7)	.51
Opioids and cocaine or methamphetamine together (n = 708)^d^	412 (58.2)	264 (65.7)	148 (48.4)	<.001
Opioids and unprescribed benzodiazepines together (n = 718)^d^	161 (22.4)	98 (24.3)	63 (20.1)	.21
Past 30-d injection drug use (n = 732)	491 (67.1)	311 (75.1)	180 (56.6)	<.001
Past 30-d overdose (n = 712)	168 (23.6)	105 (26.0)	63 (20.5)	.10
Ever received FTS instructions (n = 719)	438 (60.9)	313 (76.2)	125 (40.6)	<.001

Abbreviation: GED, General Education Development diploma.

^a^ Denotes valid number, which excludes missing responses and responses of “don’t know” or “prefer not to answer.”

^b^ Other includes American Indian or Alaska Native, Asian, Native Hawaiian or Other Pacific Islander, or some other race.

^c^ Respondents could select more than 1 drug used, so percentages do not add up to 100.

^d^ Responses “always” and “sometimes” were combined to produce the counts and percentages in this row.

When examining past 30-day drug category used and route of administration, among those reporting baseline FTS use, the prevalence of injecting drugs in the past 30 days was higher across nearly every drug category compared with those not reporting baseline FTS use (ie, illicit opioids only, illicit stimulants only, illicit opioids and illicit stimulants only, and illicit benzodiazepines) (eTable 3 in Supplement 1). Conversely, among those who reported no baseline FTS use, smoking was more prevalent across nearly all drug categories compared with those reporting baseline FTS use (eTable 3 in Supplement 1).

Baseline FTS users had a higher mean composite score of overdose risk reduction behaviors in the past 30 days and engaged in a greater mean number of these behaviors compared with nonusers (eTable 4 in Supplement 1). When examining overdose risk reduction behaviors within each baseline FTS group, FTS users reported a higher prevalence of nearly every behavior across most drug categories (eTable 5 in Supplement 1). The primary exception was noninjection drug use, which was more common among FTS nonusers across all drug categories (eTable 5 in Supplement 1). There was no significant difference in past 30-day overdose between the 2 groups (Table 2).

### Overdose Risk Reduction Behaviors

#### Mean Daily Composite Score

eFigure 1 in Supplement 1 provides the daily sample mean of the composite score (range 0-15) of overdose risk reduction behaviors on drug use days across follow-up. After adjustment, participants who used FTS at baseline had a 0.86 (95% CI, 0.34-1.38) unit higher mean daily composite score than participants that did not use FTS at baseline (mean daily composite score for FTS users, 7.37; 95% CI, 6.63-8.11; mean daily composite score for nonusers, 6.51; 95% CI, 5.83-7.19) (Table 3).

**Table 3.** Unadjusted and Adjusted Associations Between Self-Reported Baseline Fentanyl Test Strip (FTS) Use and the Mean Daily Outcome Measures Across the 28-Day Observation Period

Estimate of association	Participants, No.	Estimate (95 CI)^a^	*P* value
Composite score of overdose risk reduction behaviors^b^			
Difference in means^c^	719	0.89 (0.44-1.35)	<.001
Adjusted difference in means^d^	684	0.86 (0.34-1.38)	.001
Count of overdose risk reduction behaviors^e^			
Rate ratio^c^	719	1.12 (1.05-1.19)	<.001
Adjusted rate ratio^d^	684	1.11 (1.04-1.19)	.001
Individual overdose risk reduction behaviors			
Someone else was present when you used drugs^e^			
Risk ratio^c^	726	1.05 (1.01-1.10)	.02
Adjusted risk ratio^d^	689	1.04 (0.99-1.09	.09
Naloxone was nearby when you used drugs^e^			
Risk ratio^c^	726	1.14 (1.08-1.21)	<.001
Adjusted risk ratio^d^	690	1.10 (1.04-1.18)	.002
Test the strength of your drugs before using them^f^			
Risk ratio^c^	726	1.40 (1.23-1.58)	<.001
Adjusted risk ratio^d^	689	1.41 (1.24-1.61)	<.001
Use your drugs slowly^e^			
Risk ratio^c^	727	1.11 (1.01-1.23)	.03
Adjusted risk ratio^d^	689	1.10 (0.99-1.22)	.08
Ask someone to check on you when using drugs^f^			
Risk ratio^c^	727	1.32 (1.16-1.51)	<.001
Adjusted risk ratio^d^	690	1.26 (1.10-1.45)	.001
Smoke, snort, sniff, swallow, or anally use your drugs^f^			
Risk ratio^c^	725	0.84 (0.77-0.91)	<.001
Adjusted risk ratio^d^	688	0.86 (0.79-0.94)	.001
Throw out drugs because they were bad or not what you expected^f^			
Risk ratio^c^	727	1.27 (0.98-1.66)	.08
Adjusted risk ratio^d^	690	1.50 (1.12-2.01)	.01
Watch someone use the same drugs to see how they reacted before you used them^f^			
Risk ratio^c^	726	1.15 (1.02-1.30)	.02
Adjusted risk ratio^d^	689	1.19 (1.04-1.36)	.01
Nonfatal overdose^f^			
Risk ratio^c^	710	2.06 (1.09-3.89)	.03
Adjusted risk ratio^g^	677	1.20 (0.70-2.06)	.52

^a^ All model estimates compare FTS use at baseline with no FTS use at baseline.

^b^ Modeled using a generalized estimating equations approach with a normal distribution, identity link, and a compound symmetry working correlation structure.

^c^ Unadjusted models include FTS use at baseline as the only independent variable.

^d^ Adjusted models include the following baseline measures: FTS use, state of residence, age, self-reported race, self-reported ethnicity, sex, highest level of education completed, employment status, ever received FTS instructions, and past 30-day overdose; and the following time-varying covariates measured on each day of drug use during the observation period: drug category used, study week, and part of week.

^e^ Modeled using a generalized estimating equations approach with a Poisson distribution, log link, and a compound symmetry working correlation structure.

^f^ Modeled using a generalized estimating equations approach with a Poisson distribution, log link, and a first-order autoregressive working correlation structure.

^g^ Adjusted model for nonfatal overdose includes the following baseline measures: FTS use, state of residence, age, self-reported race, self-reported ethnicity, and sex; and the following time-varying covariates measured on each day of drug use during the observation period: drug category used, study week, part of week, composite score of overdose risk reduction behaviors, and number of times FTS were used.

#### Mean Daily Rate of Behaviors

eFigure 2 in Supplement 1 provides the daily sample mean number of overdose risk reduction behaviors reported (range 0-8) on drug use days across follow-up. After adjustment, the mean daily rate of behaviors used across follow-up was 11% higher (relative risk [RR], 1.11; 95% CI, 1.04-1.19) among baseline FTS users compared with nonusers (Table 3).

#### Mean Daily Probability of Each Behavior

eFigure 3 in Supplement 1 shows the daily sample percentage of participants who self-reported using each of the overdose risk reduction behaviors on drug use days during follow-up. Compared with those reporting no baseline FTS use, adjusted analyses indicated that baseline FTS users were more likely to report having naloxone nearby when using drugs (RR, 1.10; 95% CI, 1.04-1.18), testing the strength of drugs before using them (RR, 1.41; 95% CI, 1.24-1.61), asking someone to check on them while using (RR, 1.26; 95% CI, 1.10-1.45), throwing out drugs because they were bad or not what was expected (RR, 1.50; 95% CI, 1.12-2.01), and watching someone using the same drugs to see how they reacted before using them (RR, 1.19; 95% CI, 1.04-1.36) (Table 3). However, baseline FTS users were less likely to smoke, snort, sniff, swallow, or anally use their drugs compared with those who self-reported no baseline FTS use (RR, 0.86; 95% CI, 0.79-0.94) (Table 3). The probabilities of someone else being present when drugs were used and using drugs slowly did not differ between the 2 groups (Table 3).

### Mean Daily Risk of Self-Reported Nonfatal Overdose

eFigure 4 in Supplement 1 provides the daily sample percentage of PWUD who self-reported a nonfatal overdose on drug use days across follow-up. The unadjusted mean of the daily risk of nonfatal overdose during follow-up was 2-fold higher (RR, 2.06; 95% CI, 1.09-3.89) in those who used FTS at baseline compared with those who did not (Table 3). After adjustment, this association was no longer statistically significant (mean of daily risk for FTS users, 0.02; 95% CI, 0.01-0.03; mean of daily risk for nonusers, 0.02; 95% CI, 0.01-0.03; RR, 1.20; 95% CI, 0.70-2.06). This association was not modified by age in unadjusted analyses, but was modified by age in adjusted analyses (*P* = .02) such that the ratio of the adjusted mean daily risk of self-reported nonfatal overdose between the 2 groups decreased as age increased (eFigure 5 in Supplement 1).

## Discussion

In this cohort study, participants who used FTS at baseline engaged in more overdose risk reduction behaviors and did so more frequently during follow-up than those who did not use FTS. This association remained after adjusting for confounders. Baseline FTS use was linked to key harm reduction behaviors, including having naloxone, testing drug strength, asking someone to check on them while using, and discarding drugs that seemed bad or unexpected. Our study expands the literature by prospectively examining this association over a 4-week follow-up, suggesting that individuals who use FTS incorporate them as part of a broader set of risk reduction strategies. Despite evidence supporting FTS’s role in harm reduction, critics argue they enable fentanyl use,^37,38^ and FTS remain classified as drug paraphernalia in several states.^39^ Our findings further counter this claim, demonstrating that FTS users also engage in multiple overdose prevention behaviors. Future research will examine how FTS results influence subsequent overdose risk reduction behaviors.

Over half of our sample used FTS, a higher proportion than reported by other studies.^14^ While FTS acceptability is high among PWUD,^14^ uptake is reportedly much lower, with a median proportion of 19%.^14^ This may reflect our recruitment from sites distributing FTS, which is likely a factor in who uses FTS and who does not,^26,40^ and underscores the importance of access and availability.

Examining drug use behaviors among those who use FTS provides context for greater adoption of overdose risk reduction behaviors. At baseline, FTS users differed significantly from nonusers in drug category used and administration routes, with FTS users reporting higher use of fentanyl, heroin, methamphetamine, and opioid-stimulant combinations and past 30-day injection drug use. The widespread presence of fentanyl in the unregulated drug supply—including in nonopioid substances such as stimulants and benzodiazepines—likely contributed to the high uptake of FTS, particularly among those engaging in high-risk behaviors such as polysubstance and injection drug use. Nonetheless, FTS users also engaged in overdose risk reduction behaviors. PWUD who employ practices to reduce overdose risk likely understand the implications of their risky drug use, but simultaneously aim to mitigate associated harms.^9,41^ Rather than rely on a single method, PWUD appear to use multiple harm reduction techniques,^42^ which may collectively reduce overdose risk. Importantly, non-FTS users were more likely to report noninjection drug use (eg, smoking and snorting) than FTS users, possibly because they may be less aware of FTS or perceive themselves at lower risk of overdose.^43,44^ However, given the pervasiveness of fentanyl across the street drug supply, including substances commonly smoked or snorted like cocaine, people who do not inject drugs remain at significant opioid overdose risk. Although harm reduction agencies, particularly syringe service programs, offer a range of safer use supplies, there are often challenges with engaging people who do not inject drugs.^45,46,47,48^ The uptake of FTS among those who inject may reflect the importance of syringe service as a touch point for information about additional resources and practices^49,50^ and highlight the need to identify creative ways to engage those who do not inject drugs but are still vulnerable to fentanyl exposure.^43^

This is one of the first studies we know of to examine the individual-level association between FTS use and nonfatal overdose.^14,51,52^ Comparison of crude rates of self-reported nonfatal overdose was significantly different between baseline FTS use groups; however, after adjusting for potential confounders, there was no difference. As described previously, baseline FTS users appeared to concurrently engage in drug use behaviors that confer (eg, polysubstance) and mitigate (eg, testing strength) overdose risk, which likely explains why differences between groups did not remain after adjustment. Further, the lack of a difference in adjusted overdose risk between baseline FTS users and nonusers suggests that FTS use alone may not be sufficient to reduce overdose risk in populations with high-risk drug behaviors. FTS may be considered an intermediate step between drug preparation and use, providing information to inform drug use and overdose risk reduction behaviors.

### Strengths and Limitations

Strengths of the study include a robust sample size powered for the primary objective, relatively good retention rates, and small, consistent research teams that were pivotal in engaging with participants, fostering rapport, trust, and recognition. Conducting the study across 3 states with varying historical legal statuses regarding FTS distribution and possession enhanced generalizability.

These findings are subject to limitations. PWUD accessing harm reduction facilities are not representative of all PWUD, as they are already seeking risk mitigation resources. Lack of data on prior harm reduction engagement may have led to overestimation or underestimation of the associations. As an observational study, harm reduction messaging varied across sites, potentially influencing FTS use and risk reduction behaviors. The predominantly non-Hispanic White sample limited generalizability, and behavioral intent was not measured, preventing us from distinguishing risk-enhancing from risk-reducing behaviors. The relatively brief follow-up period limited insights into the long-term relationship between FTS use and harm reduction strategies, and self-reported measures may be subject to social desirability and recall bias. Additionally, participant anonymity prevented assessment of reasons for loss to follow-up, including whether any fatal overdoses occurred.

## Conclusions

In this study, FTS use was associated with a higher likelihood of engaging in behaviors that reduce overdose risk; however, there was no association between FTS use and the risk of nonfatal overdose. Our findings provide support for the role of FTS in a comprehensive harm reduction strategy, but suggest that FTS use alone may be insufficient to significantly reduce overdose rates, particularly among populations with complex, high-risk drug use patterns. Integrating FTS with other harm reduction strategies like naloxone distribution is critical. These new data, alongside existing evidence on FTS acceptability and use, highlight the need to consider addressing regulatory and financial barriers to FTS distribution. Future research should prioritize more diverse populations, particularly Black, Indigenous, and Latinx communities disproportionally impacted by overdose. Determining the most effective ways to distribute and promote broadscale harm reduction education and resources, including FTS, across various community settings remains critical to combating the overdose crisis.

## Notes

Supplement 1. **eMethods. **Detailed methods for the study **eTable 1.** Visit completion in the full sample, overall and by self-reported baseline FTS use **eTable 2.** Characteristics of participants in the enrolled sample, overall and by inclusion in the full sample **eTable 3.** Route of administration in the past 30 days at baseline in participants in the full sample, overall and by self-reported baseline FTS use **eTable 4.** Past 30-day overdose risk reduction behaviors reported at baseline, overall and by self-reported baseline FTS use **eTable 5. **Overdose risk reduction behaviors used in the past 30 days at baseline by drug category in participants in the full sample, overall and by self-reported baseline FTS use **eFigure 1.** Daily sample mean of the composite score during the 28-day observation period by self-reported baseline FTS use **eFigure 2. **Daily sample mean of the number of overdose risk reduction behaviors during the 28-day observation period by self-reported baseline FTS use **eFigure 3. **Daily sample percentage of participants self-reporting each overdose risk reduction behavior during the 28-day observation period by self-reported baseline FTS use **eFigure 4**. Daily sample percentage of participants who self-reported a nonfatal overdose during the 28-day observation period by self-reported baseline FTS use **eFigure 5.** Modification of the association between self-reported baseline FTS use and self-reported nonfatal overdose by age across the 28-day observation period

Supplement 2.
Data Sharing Statement
