# Supplementary material for: Fentanyl Test Strip Use and Overdose Risk Reduction Behaviors Among People Who Use Drugs
Source: JAMA Netw Open. 2025 May 13;8(5):e2510077. doi: 10.1001/jamanetworkopen.2025.10077 (PMC12076174; doi:10.1001/jamanetworkopen.2025.10077)
Supplement: Supplement 1. — eMethods. Detailed methods for the study eTable 1. Visit completion in the full sample, overall and by self-reported baseline FTS use eTable 2. Characteristics of participants in the enrolled sample, overall and by inclusion in the full sample eTable 3. Route of administration in the past 30 days at baseline in participants in the full sample, overall and by self-reported baseline FTS use eTable 4. Past 30-day overdose risk reduction behaviors reported at baseline, overall and by self-reported baseline FTS use eTable 5. Overdose risk reduction behaviors used in the past 30 days at baseline by drug category in participants in the full sample, overall and by self-reported baseline FTS use eFigure 1. Daily sample mean of the composite score during the 28-day observation period by self-reported baseline FTS use eFigure 2. Daily sample mean of the number of overdose risk reduction behaviors during the 28-day observation period by self-reported baseline FTS use eFigure 3. Daily sample percentage of participants self-reporting each overdose risk reduction behavior during the 28-day observation period by self-reported baseline FTS use eFigure 4. Daily sample percentage of participants who self-reported a nonfatal overdose during the 28-day observation period by self-reported baseline FTS use eFigure 5. Modification of the association between self-reported baseline FTS use and self-reported nonfatal overdose by age across the 28-day observation period [file jamanetwopen-e2510077-s001.pdf]

Supplemental Online Content

Vickers-Smith RA, Gelberg KH, Childerhose JE, et al. Fentanyl test strip use and overdose risk reduction behaviors among people who use drugs. *JAMA Netw Open*. 20258(5):e2510077. doi:10.1001/jamanetworkopen.2025.10077

eMethods. Detailed methods for the study

eTable 1. Visit completion in the full sample, overall and by self-reported baseline FTS use

eTable 2. Characteristics of participants in the enrolled sample, overall and by inclusion in the full sample

eTable 3. Route of administration in the past 30 days at baseline in participants in the full sample, overall and by self-reported baseline FTS use

eTable 4. Past 30-day overdose risk reduction behaviors reported at baseline, overall and by self-reported baseline FTS use

eTable 5. Overdose risk reduction behaviors used in the past 30 days at baseline by drug category in participants in the full sample, overall and by self-reported baseline FTS use

eFigure 1. Daily sample mean of the composite score during the 28-day observation period by self-reported baseline FTS use

eFigure 2. Daily sample mean of the number of overdose risk reduction behaviors during the 28-day observation period by self-reported baseline FTS use

eFigure 3. Daily sample percentage of participants self-reporting each overdose risk reduction behavior during the 28-day observation period by self-reported baseline FTS use

eFigure 4. Daily sample percentage of participants who self-reported a nonfatal overdose during the 28-day observation period by self-reported baseline FTS use

eFigure 5. Modification of the association between self-reported baseline FTS use and self-reported nonfatal overdose by age across the 28-day observation period

This supplemental material has been provided by the authors to give readers additional information about their work.

© 2025 Vickers-Smith RA et al. *JAMA Network Open*.

eMethods. Detailed methods for the study.

Study Design

The Stay Safe Study is an ancillary study of the HEALing Communities Study (HCS).[15,16] The Stay Safe Study received ethics approval from Advarra's Institutional Review Board (Pro00068304) on March 16, 2023. The Strengthening Reporting of Observational Studies in Epidemiology (STROBE) reporting guidelines were followed. The study period was August 2022-June 2024 with recruitment and data collection occurring May-December 2023.

After providing written informed consent via an electronic tablet, participants completed a baseline survey covering sociodemographic characteristics; past 30-day: drug use, FTS use, and overdose risk reduction behaviors; and completed four weekly surveys over a 28-day observation period (referred to as 'follow-up'). The weekly surveys used a modified Timeline Followback approach[17,18] to capture self-reported patterns of FTS use, drug use, overdose risk reduction behaviors, and nonfatal overdose events on each day they used drugs in the past week. Participants were compensated up to $250 for completing the screening, baseline, and ~30-minute weekly surveys. Compensation was provided after each activity, and participants received payment for surveys they started, even if they did not complete them.

Setting

Fourteen community partner organizations that distribute FTS in three states participated and hosted data collection activities at their direct service provision sites (Kentucky [KY]=5, New York [NY]=3, Ohio [OH]=6). These included a range of fixed and mobile locations that deliver harm reduction services (e.g., buildings, mobile vans, and adjacent parking lots).

Participants

Eligibility criteria included: ≥18 years of age, self-assessed ability to read and speak English proficiently, able to understand and provide consent, currently live and plan to continue

© 2025 Vickers-Smith RA et al. *JAMA Network Open*.

living in KY, NY or OH during follow-up, and report past 30-day drug use (see Variables for drug list). Figure 1 summarizes those screened, eligible, enrolled, and followed during the study. To be considered enrolled, participants were required to complete the ~30-minute baseline survey; completion of a survey was defined as answering at least one question. To be included in the analytic sample (referred to as the 'Full Sample'), participants had to have self-reported drug use on ≥ one day and completed ≥ two weekly surveys during follow-up.

Variables and Measurement

Outcomes

Overdose risk reduction behaviors. The primary outcome was a composite score measuring the number and frequency of eight overdose risk reduction behaviors on each day drugs were used (Table 1). These behaviors were (a) commonly recommended in the literature to reduce overdose risk, and (b) independent from one another. Participants indicated whether they always, sometimes, or never engaged in each behavior that day, and scored accordingly (Table 1). The daily composite score summed frequencies across all eight overdose risk reduction behaviors, ranging from 0 (i.e., did not engage in any behaviors that day) to 15 (i.e., engaged in all behaviors every time drugs were used that day). One behavior, "threw out the drugs because they were bad or not what you expected," could only contribute 0 or 1 to the score because if a participant always discarded drugs, then they would not have used any drugs that day (Table 1).

On each drug use day, we analyzed the following secondary outcomes: (1) the overall number and (2) each individual behavior reported. For these analyses, responses of 'always' or 'sometimes' collapsed into a single 'yes' category, contributing to the behavior count for that day.

© 2025 Vickers-Smith RA et al. *JAMA Network Open*.

Nonfatal overdose. This secondary outcome was collected via self-report in the four weekly surveys. On each drug use day, participants were prompted to report a time in the past week "when they lost consciousness, and someone had to do something to revive them".

Exposure

Baseline FTS use. The primary exposure was self-reported past 30-day FTS use (yes/no) measured at baseline. Individuals who did not respond, or indicated "don't know" or "prefer not to answer" were categorized as not using FTS.

Covariates

Covariates included in the model for each outcome were pre-specified prior to analysis based on study design and expert opinion.

Sociodemographic and drug use characteristics. Sociodemographic characteristics were self-reported on the baseline survey including: state of residence (KY, NY, or OH), age (years), race (Black or African American, white, other [American Indian or Alaska Native, Asian, Native Hawaiian or Other Pacific Islander, or some other race]), ethnicity (Hispanic or Latino, not Hispanic or Latino), sex (male, female), highest level of education completed (less than high school, high school graduate/GED or equivalent, more than high school [some college but no degree, associate degree, bachelor's degree, post-college/graduate degree, other]), employment status (employed [full-time or part-time], unemployed [temporarily laid off, unemployed and looking, unemployed and not looking], other [retired, homemaker, disabled, student, other]), ever received FTS instructions (yes, no), and past 30-day overdose (yes, no). Given that overdose rates are declining overall but increasing among certain racial and ethnic groups,[19] we included race and ethnicity separately in our analyses to account for

© 2025 Vickers-Smith RA et al. *JAMA Network Open*.

differential risk due to unmeasured factors such as unequal access to care and structural barriers. We collected both sex at birth and gender identity at baseline; we chose to include only sex in analyses because of the differential overdose risk likely conferred by biological sex[20] and high concordance with gender in our study.

Characteristics varying over time. Characteristics were self-reported on a daily basis in the weekly surveys and included: study week (1, 2, 3, or 4), part of week (weekday or weekend), number of times FTS were used, injection drug use (yes/no), and drug category used. Questions on drug use included heroin, fentanyl, powder cocaine, crack or rock cocaine, methamphetamine, “pain pills” or opioids not prescribed for participant (such as Vicodin, Percocet, hydrocodone, oxycodone), benzodiazepines not prescribed for participant (such as Valium, Xanax, or Klonopin), and stimulants not prescribed for participant (such as Ritalin or Adderall). Drug category used was then defined by collapsing responses to these questions into five mutually exclusive categories: illicit opioids only (includes heroin, fentanyl, and “pain pills” or opioids not prescribed for participant), illicit stimulants only (includes powder cocaine, crack or rock cocaine, methamphetamine, and stimulants not prescribed for participant), illicit opioids and illicit stimulants only (includes reports of illicit stimulants and illicit opioids), illicit benzodiazepines (with or without illicit opioids and/or illicit stimulants, and includes benzodiazepines not prescribed for participant), or no drug use/don't know/prefer not to answer.

Statistical Analysis

We calculated descriptive statistics for baseline characteristics in the Full Sample and compared them between participants with baseline FTS use versus no baseline FTS use using a Pearson’s chi-squared test or Fisher’s exact test for nominal/categorical variables and a Wilcoxon Rank Sum test for continuous variables. Population-averaged estimates and 95%

© 2025 Vickers-Smith RA et al. *JAMA Network Open*.

confidence intervals (CIs) of the association between baseline FTS use and each outcome were assessed with generalized estimating equations (GEE) approach with an appropriate distribution (i.e., Normal or Poisson) and link function (i.e., identity or log). Each model included baseline FTS use as the primary exposure, pre-specified baseline covariates (state of residence, age, self-reported race, self-reported ethnicity, sex, and for overdose risk reduction behavior models only: [highest level of education completed, employment status, ever received FTS instructions, past 30-day overdose]), and pre-specified time-varying covariates (drug category used, study week, part of the week; daily composite score and number of times FTS were used were also included as covariates for the nonfatal overdose model only). The covariance structure for residual effects was selected by comparing information criteria across models with different covariance structures and selecting the structure that produced the best fit. We tested whether the association between baseline FTS use and the composite score (and self-reported nonfatal overdose) was modified by pre-specified variables (state of residence, age, self-reported race, self-reported ethnicity, sex, study week, and daily injection drug use [for the nonfatal overdose model only]) by including a two-way interaction between baseline FTS use and each variable. Because the GEE approach assumes data are missing completely at random, a generalized linear mixed model that assumes data are missing at random was fit to the composite score to assess potential bias arising from missing data; the difference between the models was negligible. All hypotheses were tested using a two-sided significance level of 0.05. Consequently, all p-values presented are purely descriptive. All analyses were conducted using SAS Version 9.4 and R Version 4.3.3.

© 2025 Vickers-Smith RA et al. *JAMA Network Open*.

eTable 1: Visit completion in the Full Sample[1], overall and by self-reported baseline FTS use.

Characteristic	FTS use at baseline[2]	No FTS use at baseline[3]	Full Sample[1]
Enrolled participants in the Full Sample[1], n	414	318	732
Completed[4] 1st weekly survey, n (% enrolled)	343 (82.9)	267 (84.0)	610 (83.3)
Completed[4] 2nd weekly survey, n (% enrolled)	366 (88.4)	286 (89.9)	652 (89.1)
Withdrew between 2nd and 3rd weekly survey, n (% enrolled)			
Lost to follow-up	11 (2.7)	6 (1.9)	17 (2.3)
Completed[4] 3rd weekly survey, n (% enrolled)	373 (90.1)	292 (91.8)	665 (90.8)
Withdrew between 3rd and 4th weekly survey, n (% enrolled)			
Lost to follow-up	40 (9.7)	27 (8.5)	67 (9.2)
Completed[4] 4th weekly survey, n (% enrolled)	352 (85.0)	274 (86.2)	626 (85.5)

[1]The Full Sample includes all participants in the Enrolled Sample who: 1) completed at least two of the Weekly Surveys; and 2) reported drug use at least once during the 28-day observation period. Completion of a survey is defined by the presence of any data in the survey.
[2]Participants with self-reported FTS use at baseline includes all individuals in the Full Sample who answered 'Yes' to question the Baseline Survey question: In the past 30 days, have you used fentanyl test strips to check your drugs?
[3]Participants with self-reported no FTS use at baseline includes all individuals in the Full Sample who do not meet the definition of self-reported FTS use at baseline.
[4]Completion of a survey is defined by the presence of any data in the survey.

© 2025 Vickers-Smith RA et al. *JAMA Network Open*.

eTable 2. Characteristics of participants in the Enrolled Sample, overall and by inclusion in the Full Sample.

Characteristic	Enrolled Sample[1]	Full Sample[2]	Not in Full Sample[2]	p-value
Number of participants in the Enrolled Sample[1], n	(N=1156)	(N=732)	(N=424)	
State of residence, n/N (%)				
Kentucky	327/1156 (28.3)	254/732 (34.7)	73/424 (17.2)	<0.001
New York	345/1156 (29.8)	162/732 (22.1)	183/424 (43.2)	
Ohio	484/1156 (41.9)	316/732 (43.2)	168/424 (39.6)	
Age (years)				
Median (1st quartile, 3rd quartile)	40.0 (34.0-48.0)	41.0 (34.0-48.0)	40.0 (33.0-47.0)	0.03
Don't know, n/N[3] (%)	1/1153 (0.1)	0 (0.0)	1/421 (0.2)	0.37
Prefer not to answer, n/N[3] (%)	3/1153 (0.3)	0 (0.0)	3/421 (0.7)	0.05
Missing, n	3	0	3	NA
Self-reported race[4], n/N[3] (%)				
Black or African American	111/1145 (9.7)	64/732 (8.7)	47/413 (11.4)	<0.001
White	869/1145 (75.9)	587/732 (80.2)	282/413 (68.3)	
Other	142/1145 (12.4)	71/732 (9.7)	71/413 (17.2)	
Don't know	8/1145 (0.7)	5/732 (0.7)	3/413 (0.7)	
Prefer not to answer	15/1145 (1.3)	5/732 (0.7)	10/413 (2.4)	
Missing, n	11	0	11	
Self-reported ethnicity[5], n/N[3] (%)				
Hispanic or Latino	145/1148 (12.6)	64/732 (8.7)	81/416 (19.5)	<0.001
Not Hispanic or Latino	990/1148 (86.2)	660/732 (90.2)	330/416 (79.3)	
Don't know	6/1148 (0.5)	4/732 (0.5)	2/416 (0.5)	
Prefer not to answer	7/1148 (0.6)	4/732 (0.5)	3/416 (0.7)	
Missing, n	8	0	8	
Sex, n/N[3] (%)				
Female	553/1145 (48.3)	363/732 (49.6)	190/413 (46.0)	0.01
Male	587/1145 (51.3)	369/732 (50.4)	218/413 (52.8)	
Don't know	1/1145 (0.1)	0 (0.0)	1/413 (0.2)	

© 2025 Vickers-Smith RA et al. *JAMA Network Open*.

Characteristic	Enrolled Sample[1]	Full Sample[2]	Not in Full Sample[2]	p-value
Number of participants in the Enrolled Sample[1], n	(N=1156)	(N=732)	(N=424)	
Prefer not to answer	4/1145 (0.3)	0 (0.0)	4/413 (1.0)	
Missing, n	11	0	11	
Highest level of education completed, n/N[3] (%)				
Primary (less than high school)	123/1140 (10.8)	73/732 (10.0)	50/408 (12.3)	0.27
Secondary (some high school, high school graduate, or GED)	634/1140 (55.6)	417/732 (57.0)	217/408 (53.2)	
Post-secondary (some college or post-secondary degree)	367/1140 (32.2)	235/732 (32.1)	132/408 (32.4)	
Don't know	5/1140 (0.4)	2/732 (0.3)	3/408 (0.7)	
Prefer not to answer	11/1140 (1.0)	5/732 (0.7)	6/408 (1.5)	
Missing, n	16	0	16	
Employment status, n/N[3] (%)				
Unemployed	544/1117 (48.7)	391/732 (53.4)	153/385 (39.7)	<0.001
Employed (e.g., part-time or full-time)	298/1117 (26.7)	169/732 (23.1)	129/385 (33.5)	
Other (e.g., retired, disabled, student, homemaker)	237/1117 (21.2)	155/732 (21.2)	82/385 (21.3)	
Don't know	13/1117 (1.2)	5/732 (0.7)	8/385 (2.1)	
Prefer not to answer	25/1117 (2.2)	12/732 (1.6)	13/385 (3.4)	
Missing, n	39	0	39	

[1]The Enrolled Sample includes all eligible participants who completed the Baseline Survey. Completion of a survey is defined by the presence of any data in the survey.
[2]The Full Sample includes all participants in the Enrolled Sample who: 1) completed at least two of the weekly surveys; and 2) reported drug use at least once during the 28-day observation period. Completion of a survey is defined by the presence of any data in the survey.
[3]Number of participants who have a non-missing response to the question measuring this characteristic.
[4]Race was by captured by self-report in response to the following question: "What is your race? Check all that apply." Options included: American Indian or Alaska Native; 2) Asian; 3) Black or African American; 4) Native Hawaiian or Other Pacific Islander; 5) White; 6) Other (Please specify); 7) Don't know; or 8); Prefer not to answer. The following options were included in 'Other' category: 1) American Indian or Alaska Native; 2) Asian; 3) Native Hawaiian or Other Pacific Islander; 4) Other (Please specify).
[5]Ethnicity was by captured by self-report in response to the following question: "Do you consider yourself to be Hispanic or Latino?" Options included: 1) Yes; 2) No; 3) Don't know; or 4); Prefer not to answer.

© 2025 Vickers-Smith RA et al. *JAMA Network Open*.

eTable 3: Route of administration in the past 30 days at baseline in participants in the Full Sample[1], overall and by self-reported baseline FTS use.

Characteristic	Full Sample[1]	Self-reported FTS use at baseline[2]	Self-reported no FTS use at baseline[3]	p-value
Number of participants in the Full Sample[1], n	(N=732)	(N=414)	(N=318)	
Type of drugs used in the past 30 days, n/N[4] (%)				
Illicit opioids only	71/732(9.70)	38/414(9.18)	33/318(10.38)	0.0003[10]
Illicit stimulants only	98/732(13.39)	39/414(9.42)	59/318(18.55)	
Illicit opioids and illicit stimulants only	318/732(43.44)	198/414(47.83)	120/318(37.74)	
Illicit benzodiazepines (with or without illicit opioids, with or without illicit stimulants)	189/732(25.82)	115/414(27.78)	74/318(23.27)	
No drug use/don't know/prefer not to answer	56/732(7.65)	24/414(5.80)	32/318(10.06)	
Drugs were swallowed in the past 30 days, n/N[4] (%)	234/732(31.97)	129/414(31.16)	105/318(33.02)	
Illicit opioids only, n/N[5] (%)	16/71(22.54)	5/38(13.16)	11/33(33.33)	< 0.001[11]
Illicit stimulants only, n/N[6] (%)	13/98(13.27)	5/39(12.82)	8/59(13.56)	
Illicit opioids and illicit stimulants only, n/N[7] (%)	64/318(20.13)	42/198(21.21)	22/120(18.33)	
Illicit benzodiazepines (with or without illicit opioids, with or without illicit stimulants), n/N[8] (%)	128/189(67.72)	72/115(62.61)	56/74(75.68)	
No drug use/don't know/prefer not to answer, n/N[9] (%)	13/56(23.21)	5/24(20.83)	8/32(25.00)	
Drugs were snorted or sniffed in the past 30 days, n/N[4] (%)	421/732(57.51)	222/414(53.62)	199/318(62.58)	
Illicit opioids only, n/N[5] (%)	24/71(33.80)	13/38(34.21)	11/33(33.33)	< 0.001[11]
Illicit stimulants only, n/N[6] (%)	50/98(51.02)	17/39(43.59)	33/59(55.93)	
Illicit opioids and illicit stimulants only, n/N[7] (%)	167/318(52.52)	95/198(47.98)	72/120(60.00)	
Illicit benzodiazepines (with or without illicit opioids, with or without illicit stimulants), n/N[8] (%)	146/189(77.25)	84/115(73.04)	62/74(83.78)	
No drug use/don't know/prefer not to answer, n/N[9] (%)	34/56(60.71)	13/24(54.17)	21/32(65.63)	
Drugs were smoked in the past 30 days, n/N[4] (%)	470/732(64.21)	259/414(62.56)	211/318(66.35)	
Illicit opioids only, n/N[5] (%)	7/71(9.86)	3/38(7.89)	4/33(12.12)	< 0.001[11]

© 2025 Vickers-Smith RA et al. *JAMA Network Open*.

Illicit stimulants only, n/N[6] (%)	67/98(68.37)	23/39(58.97)	44/59(74.58)	
Illicit opioids and illicit stimulants only, n/N[7] (%)	219/318(68.87)	132/198(66.67)	87/120(72.50)	
Illicit benzodiazepines (with or without illicit opioids, with or without illicit stimulants), n/N[8] (%)	139/189(73.54)	82/115(71.30)	57/74(77.03)	
No drug use/don't know/prefer not to answer, n/N[9] (%)	38/56(67.86)	19/24(79.17)	19/32(59.38)	
Drugs were injected in the past 30 days, n/N[4] (%)	491/732(67.08)	311/414(75.12)	180/318(56.60)	
Illicit opioids only, n/N[5] (%)	49/71(69.01)	31/38(81.58)	18/33(54.55)	< 0.001[11]
Illicit stimulants only, n/N[6] (%)	39/98(39.80)	22/39(56.41)	17/59(28.81)	
Illicit opioids and illicit stimulants only, n/N[7] (%)	237/318(74.53)	159/198(80.30)	78/120(65.00)	
Illicit benzodiazepines (with or without illicit opioids, with or without illicit stimulants), n/N[8] (%)	135/189(71.43)	86/115(74.78)	49/74(66.22)	
No drug use/don't know/prefer not to answer, n/N[9] (%)	31/56(55.36)	13/24(54.17)	18/32(56.25)	
Drugs were used anally (plugged or boofed) in the past 30 days, n/N[4] (%)	29/732(3.96)	16/414(3.86)	13/318(4.09)	
Illicit opioids only, n/N[5] (%)	1/71(1.41)	0/38(0.00)	1/33(3.03)	0.4476[11]
Illicit stimulants only, n/N[6] (%)	4/98(4.08)	1/39(2.56)	3/59(5.08)	
Illicit opioids and illicit stimulants only, n/N[7] (%)	12/318(3.77)	10/198(5.05)	2/120(1.67)	
Illicit benzodiazepines (with or without illicit opioids, with or without illicit stimulants), n/N[8] (%)	11/189(5.82)	5/115(4.35)	6/74(8.11)	
No drug use/don't know/prefer not to answer, n/N[9] (%)	1/56(1.79)	0/24(0.00)	1/32(3.13)	

[1]The Full Sample includes all PWUD in the Enrolled Sample who: 1) completed at least two of the Weekly Surveys; and 2) reported drug use at least once during the 28-day observation period. Completion of a survey is defined by the presence of any data in the survey.
[2]Participants with self-reported FTS use at baseline includes all individuals in the Full Sample who answered 'Yes' to the following Baseline Survey question: In the past 30 days, have you used fentanyl test strips to check your drugs?
[3]PWUD with self-reported non-FTS use at baseline includes all PWUD in the Full Sample who do not meet the definition of self-reported FTS use at baseline.
[4]Number of PWUD who have a non-missing response to the question measuring this characteristic.
[5]Number of PWUD who used illicit opioids only.
[6]Number of PWUD who used illicit stimulants only.
[7]Number of PWUD who used illicit opioids and illicit stimulants only.
[8]Number of PWUD who used illicit benzodiazepines (with or without illicit opioids, with or without illicit stimulants)
[9]Number of PWUD who did not use drugs or answered don't know or prefer to not answer.
[10]P-value obtained from a Pearson chi-squared test.
[11]P-value obtained from a Mantel-Haenszel test, stratified by self-reported FTS use at baseline.

© 2025 Vickers-Smith RA et al. *JAMA Network Open*.

eTable 4: Past 30-day overdose risk reduction behaviors reported at baseline in the Full Sample, overall and by self-reported baseline FTS use.

Characteristic	Full Sample[1]	FTS use at baseline[2]	No FTS use at baseline[3]	p-value
Number of participants in the Full Sample[1] who used drugs in the past 30 days, n	(N=726)	(N=414)	(N=312)	
Composite score of overdose risk reduction behaviors				
Median (1st quartile, 3rd quartile)	8.0 (6.0-10.0)	8.0 (7.0-10.0)	7.0 (5.0-8.0)	<0.001
Missing, n	112	60	52	NA
Number of overdose risk reduction behaviors used				
Median (1st quartile, 3rd quartile)	6.0 (4.0-7.0)	6.0 (5.0-7.0)	5.0 (4.0-6.0)	<0.001
Missing, n	112	60	52	NA
Someone else present when you used drugs, n/N[4] (%)				
Always	356/726 (49.0)	221/414 (53.4)	135/312 (43.3)	<0.001
Sometimes	335/726 (46.1)	182/414 (44.0)	153/312 (49.0)	
Never	32/726 (4.4)	9/414 (2.2)	23/312 (7.4)	
Don't know	2/726 (0.3)	1/414 (0.2)	1/312 (0.3)	
Prefer not to answer	1/726 (0.1)	1/414 (0.2)	0 (0.0)	
Missing, n	0	0	0	
Naloxone was nearby when you used drugs, n/N[4] (%)				
Always	388/726 (53.4)	267/414 (64.5)	121/312 (38.8)	<0.001
Sometimes	205/726 (28.2)	93/414 (22.5)	112/312 (35.9)	
Never	118/726 (16.3)	48/414 (11.6)	70/312 (22.4)	
Don't know	14/726 (1.9)	6/414 (1.4)	8/312 (2.6)	
Prefer not to answer	1/726 (0.1)	0 (0.0)	1/312 (0.3)	
Missing, n	0	0	0	
Test the strength of your drugs before using them, n/N[4] (%)				
Always	204/726 (28.1)	151/414 (36.5)	53/312 (17.0)	<0.001
Sometimes	284/726 (39.1)	196/414 (47.3)	88/312 (28.2)	
Never	229/726 (31.5)	61/414 (14.7)	168/312 (53.8)	

© 2025 Vickers-Smith RA et al. *JAMA Network Open*.

Characteristic	Full Sample[1]	FTS use at baseline[2]	No FTS use at baseline[3]	p-value
Number of participants in the Full Sample[1] who used drugs in the past 30 days, n	(N=726)	(N=414)	(N=312)	
Don't know	4/726 (0.6)	2/414 (0.5)	2/312 (0.6)	
Prefer not to answer	5/726 (0.7)	4/414 (1.0)	1/312 (0.3)	
Missing, n	0	0	0	
Use your drugs slowly, n/N[4] (%)				
Always	199/726 (27.4)	137/414 (33.1)	62/312 (19.9)	<0.001
Sometimes	348/726 (47.9)	208/414 (50.2)	140/312 (44.9)	
Never	171/726 (23.6)	66/414 (15.9)	105/312 (33.7)	
Don't know	5/726 (0.7)	2/414 (0.5)	3/312 (1.0)	
Prefer not to answer	3/726 (0.4)	1/414 (0.2)	2/312 (0.6)	
Missing, n	0	0	0	
Ask someone to check on you when you were using, n/N[4] (%)				
Always	142/726 (19.6)	113/414 (27.3)	29/312 (9.3)	<0.001
Sometimes	233/726 (32.1)	139/414 (33.6)	94/312 (30.1)	
Never	338/726 (46.6)	156/414 (37.7)	182/312 (58.3)	
Don't know	10/726 (1.4)	5/414 (1.2)	5/312 (1.6)	
Prefer not to answer	3/726 (0.4)	1/414 (0.2)	2/312 (0.6)	
Missing, n	0	0	0	
Smoke, snort, sniff, swallow, or anally use your drugs, n/N[4] (%)				
Always	263/725 (36.3)	134/414 (32.4)	129/311 (41.5)	0.008
Sometimes	292/725 (40.3)	168/414 (40.6)	124/311 (39.9)	
Never (only inject)	103/725 (14.2)	74/414 (17.9)	29/311 (9.3)	
Don't know	45/725 (6.2)	27/414 (6.5)	18/311 (5.8)	
Prefer not to answer	22/725 (3.0)	11/414 (2.7)	11/311 (3.5)	
Missing, n	1	0	1	
Throw out drugs because they were bad or not what you expected, n/N[4] (%)				
Sometimes	189/726 (26.0)	122/414 (29.5)	67/312 (21.5)	0.04
Never	518/726 (71.3)	281/414 (67.9)	237/312 (76.0)	

© 2025 Vickers-Smith RA et al. *JAMA Network Open*.

Characteristic	Full Sample[1]	FTS use at baseline[2]	No FTS use at baseline[3]	p-value
Number of participants in the Full Sample[1] who used drugs in the past 30 days, n	(N=726)	(N=414)	(N=312)	
Don't know	17/726 (2.3)	9/414 (2.2)	8/312 (2.6)	
Prefer not to answer	2/726 (0.3)	2/414 (0.5)	0 (0.0)	
Missing, n	0	0	0	
Watch someone use the same drugs to see how they reacted before you used them, n/N[4] (%)				
Always	93/726 (12.8)	65/414 (15.7)	28/312 (9.0)	0.003
Sometimes	323/726 (44.5)	193/414 (46.6)	130/312 (41.7)	
Never	300/726 (41.3)	153/414 (37.0)	147/312 (47.1)	
Don't know	6/726 (0.8)	2/414 (0.5)	4/312 (1.3)	
Prefer not to answer	4/726 (0.6)	1/414 (0.2)	3/312 (1.0)	
Missing, n	0	0	0	

[1]The Full Sample includes all participants in the Enrolled Sample who: 1) completed at least two of the weekly surveys; and 2) reported drug use at least once during the 28-day observation period. Completion of a survey is defined by the presence of any data in the survey.
[2]Participants with self-reported FTS use at baseline includes all individuals in the Full Sample who answered 'Yes' to the following Baseline Survey question: In the past 30 days, have you used fentanyl test strips to check your drugs?
[3]Participants with self-reported no FTS use at baseline includes all individuals in the Full Sample who do not meet the definition of self-reported FTS use at baseline.
[4]Number of participants who have a non-missing response to the question measuring this characteristic.

© 2025 Vickers-Smith RA et al. *JAMA Network Open*.

eTable 5: Overdose risk reduction behaviors used in the past 30 days at baseline by drug category in participants in the Full Sample[1], overall and by self-reported baseline FTS use.

Characteristic	Full Sample[1]	Self-reported FTS use at baseline[2]	Self-reported no FTS use at baseline[3]	p-value
Number of participants in the Full Sample[1], n	(N=732)	(N=414)	(N=318)	
Type of drugs used in the past 30 days, n/N[4] (%)				
Illicit opioids only	71/732(9.70)	38/414(9.18)	33/318(10.38)	0.0003[10]
Illicit stimulants only	98/732(13.39)	39/414(9.42)	59/318(18.55)	
Illicit opioids and illicit stimulants only	318/732(43.44)	198/414(47.83)	120/318(37.74)	
Illicit benzodiazepines (with or without illicit opioids, with or without illicit stimulants)	189/732(25.82)	115/414(27.78)	74/318(23.27)	
No drug use/don't know/prefer not to answer	56/732(7.65)	24/414(5.80)	32/318(10.06)	
Someone else present when you used drugs in the past 30 days, n/N[4] (%)	694/728(95.33)	403/412(97.82)	291/316(92.09)	
Illicit opioids only, n/N[5] (%)	62/71(87.32)	36/38(94.74)	26/33(78.79)	<0.001[11]
Illicit stimulants only, n/N[6] (%)	90/98(91.84)	39/39(100.00)	51/59(86.44)	
Illicit opioids and illicit stimulants only, n/N[7] (%)	307/317(96.85)	192/197(97.46)	115/120(95.83)	
Illicit benzodiazepines (with or without illicit opioids, with or without illicit stimulants), n/N[8] (%)	188/189(99.47)	114/115(99.13)	74/74(100.00)	
No drug use/don't know/prefer not to answer, n/N[9] (%)	47/53(88.68)	22/23(95.65)	25/30(83.33)	
Naloxone (Narcan) was nearby when you used drugs in the past 30 days, n/N[4] (%)	596/716(83.24)	360/408(88.24)	236/308(76.62)	
Illicit opioids only, n/N[5] (%)	58/71(81.69)	33/38(86.84)	25/33(75.76)	<0.001[11]
Illicit stimulants only, n/N[6] (%)	65/97(67.01)	34/39(87.18)	31/58(53.45)	
Illicit opioids and illicit stimulants only, n/N[7] (%)	263/311(84.57)	170/195(87.18)	93/116(80.17)	
Illicit benzodiazepines (with or without illicit opioids, with or without illicit stimulants), n/N[8] (%)	165/184(89.67)	101/113(89.38)	64/71(90.14)	
No drug use/don't know/prefer not to answer, n/N[9] (%)	45/53(84.91)	22/23(95.65)	23/30(76.67)	
Test the strength of your drugs before using them in the past 30 days, n/N[4] (%)	489/723(67.63)	347/408(85.05)	142/315(45.08)	
Illicit opioids only, n/N[5] (%)	43/71(60.56)	32/38(84.21)	11/33(33.33)	0.0026[11]

© 2025 Vickers-Smith RA et al. *JAMA Network Open*.

Illicit stimulants only, n/N[6] (%)	47/97(48.45)	25/39(64.10)	22/58(37.93)	
Illicit opioids and illicit stimulants only, n/N[7] (%)	230/315(73.02)	173/196(88.27)	57/119(47.90)	
Illicit benzodiazepines (with or without illicit opioids, with or without illicit stimulants), n/N[8] (%)	139/185(75.14)	96/112(85.71)	43/73(58.90)	
No drug use/don't know/prefer not to answer, n/N[9] (%)	30/55(54.55)	21/23(91.30)	9/32(28.13)	
Use your drugs slowly in the past 30 days, n/N[4] (%)	549/724(75.83)	345/411(83.94)	204/313(65.18)	
Illicit opioids only, n/N[5] (%)	47/71(66.20)	31/38(81.58)	16/33(48.48)	
Illicit stimulants only, n/N[6] (%)	58/97(59.79)	25/39(64.10)	33/58(56.90)	
Illicit opioids and illicit stimulants only, n/N[7] (%)	251/317(79.18)	167/198(84.34)	84/119(70.59)	0.0018[11]
Illicit benzodiazepines (with or without illicit opioids, with or without illicit stimulants), n/N[8] (%)	153/185(82.70)	99/112(88.39)	54/73(73.97)	
No drug use/don't know/prefer not to answer, n/N[9] (%)	40/54(74.07)	23/24(95.83)	17/30(56.67)	
Ask someone to check on you when you were using in the past 30 days, n/N[4] (%)	376/719(52.29)	252/408(61.76)	124/311(39.87)	
Illicit opioids only, n/N[5] (%)	35/69(50.72)	26/37(70.27)	9/32(28.13)	
Illicit stimulants only, n/N[6] (%)	28/98(28.57)	13/39(33.33)	15/59(25.42)	
Illicit opioids and illicit stimulants only, n/N[7] (%)	183/316(57.91)	130/197(65.99)	53/119(44.54)	<0.001[11]
Illicit benzodiazepines (with or without illicit opioids, with or without illicit stimulants), n/N[8] (%)	101/183(55.19)	68/111(61.26)	33/72(45.83)	
No drug use/don't know/prefer not to answer, n/N[9] (%)	29/53(54.72)	15/24(62.50)	14/29(48.28)	
Smoke, snort, sniff, swallow, or anally use your drugs in the past 30 days, n/N[4] (%)	555/658(84.35)	302/376(80.32)	253/282(89.72)	
Illicit opioids only, n/N[5] (%)	31/65(47.69)	13/34(38.24)	18/31(58.06)	
Illicit stimulants only, n/N[6] (%)	79/90(87.78)	30/37(81.08)	49/53(92.45)	
Illicit opioids and illicit stimulants only, n/N[7] (%)	241/290(83.10)	141/179(78.77)	100/111(90.09)	<0.001[11]
Illicit benzodiazepines (with or without illicit opioids, with or without illicit stimulants), n/N[8] (%)	164/170(96.47)	99/105(94.29)	65/65(100.00)	
No drug use/don't know/prefer not to answer, n/N[9] (%)	40/43(93.02)	19/21(90.48)	21/22(95.45)	
Throw out drugs because they were bad or not what you expected in the past 30 days, n/N[4] (%)	191/713(26.79)	122/403(30.27)	69/310(22.26)	
Illicit opioids only, n/N[5] (%)	22/69(31.88)	16/38(42.11)	6/31(19.35)	0.0183[11]

© 2025 Vickers-Smith RA et al. *JAMA Network Open*.

Illicit stimulants only, n/N[6] (%)	18/97(18.56)	6/38(15.79)	12/59(20.34)	
Illicit opioids and illicit stimulants only, n/N[7] (%)	73/313(23.32)	51/194(26.29)	22/119(18.49)	
Illicit benzodiazepines (with or without illicit opioids, with or without illicit stimulants), n/N[8] (%)	59/183(32.24)	38/112(33.93)	21/71(29.58)	
No drug use/don't know/prefer not to answer, n/N[9] (%)	19/51(37.25)	11/21(52.38)	8/30(26.67)	
Watch someone use the same drugs to see how they reacted before you used them in the past 30 days, n/N[4] (%)	419/722(58.03)	258/411(62.77)	161/311(51.77)	
Illicit opioids only, n/N[5] (%)	46/70(65.71)	29/38(76.32)	17/32(53.13)	
Illicit stimulants only, n/N[6] (%)	43/97(44.33)	15/39(38.46)	28/58(48.28)	
Illicit opioids and illicit stimulants only, n/N[7] (%)	173/314(55.10)	118/196(60.20)	55/118(46.61)	0.0066[11]
Illicit benzodiazepines (with or without illicit opioids, with or without illicit stimulants), n/N[8] (%)	123/186(66.13)	80/114(70.18)	43/72(59.72)	
No drug use/don't know/prefer not to answer, n/N[9] (%)	34/55(61.82)	16/24(66.67)	18/31(58.06)	

[1]The Full Sample includes all PWUD in the Enrolled Sample who: 1) completed at least two of the Weekly Surveys; and 2) reported drug use at least once during the 28-day observation period. Completion of a survey is defined by the presence of any data in the survey.
[2]Participants with self-reported FTS use at baseline includes all individuals in the Full Sample who answered 'Yes' to the following Baseline Survey question: In the past 30 days, have you used fentanyl test strips to check your drugs?
[3]PWUD with self-reported non-FTS use at baseline includes all PWUD in the Full Sample who do not meet the definition of self-reported FTS use at baseline.
[4]Number of PWUD who have a non-missing response to the question measuring this characteristic.
[5]Number of PWUD who used illicit opioids only.
[6]Number of PWUD who used illicit stimulants only.
[7]Number of PWUD who used illicit opioids and illicit stimulants only.
[8]Number of PWUD who used illicit benzodiazepines (with or without illicit opioids, with or without illicit stimulants)
[9]Number of PWUD who did not use drugs or answered don't know or prefer to not answer.
[10]P-value obtained from a Pearson chi-squared test.
[11]P-value obtained from a Mantel-Haenszel test, stratified by self-reported FTS use at baseline.

© 2025 Vickers-Smith RA et al. *JAMA Network Open*.

eFigure 1: Daily sample mean of the composite score during the 28-day observation period by self-reported baseline FTS use.

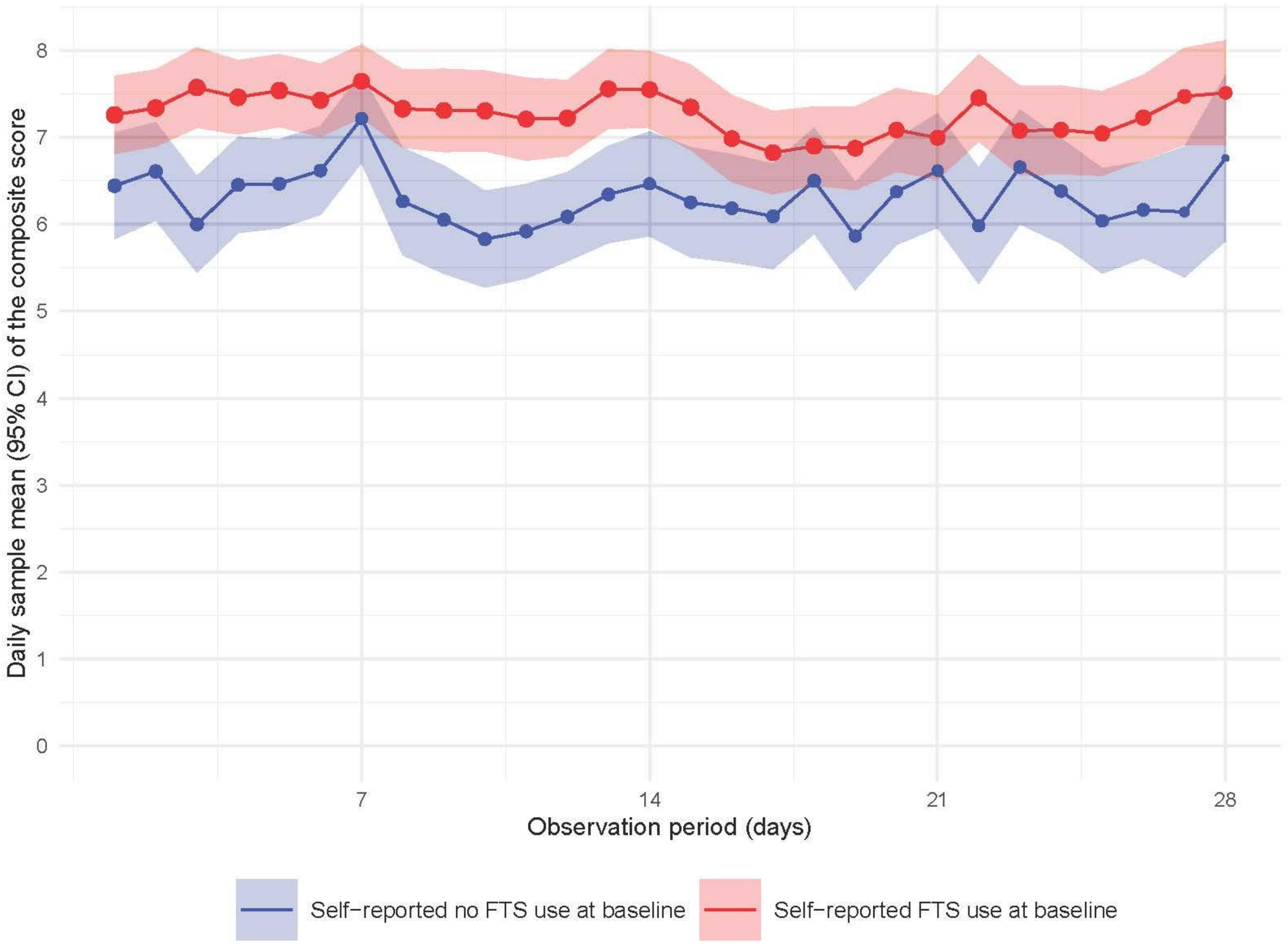

© 2025 Vickers-Smith RA et al. *JAMA Network Open*.

eFigure 2: Daily sample mean of the number of overdose risk reduction behaviors during the 28-day observation period by self-reported baseline FTS use.

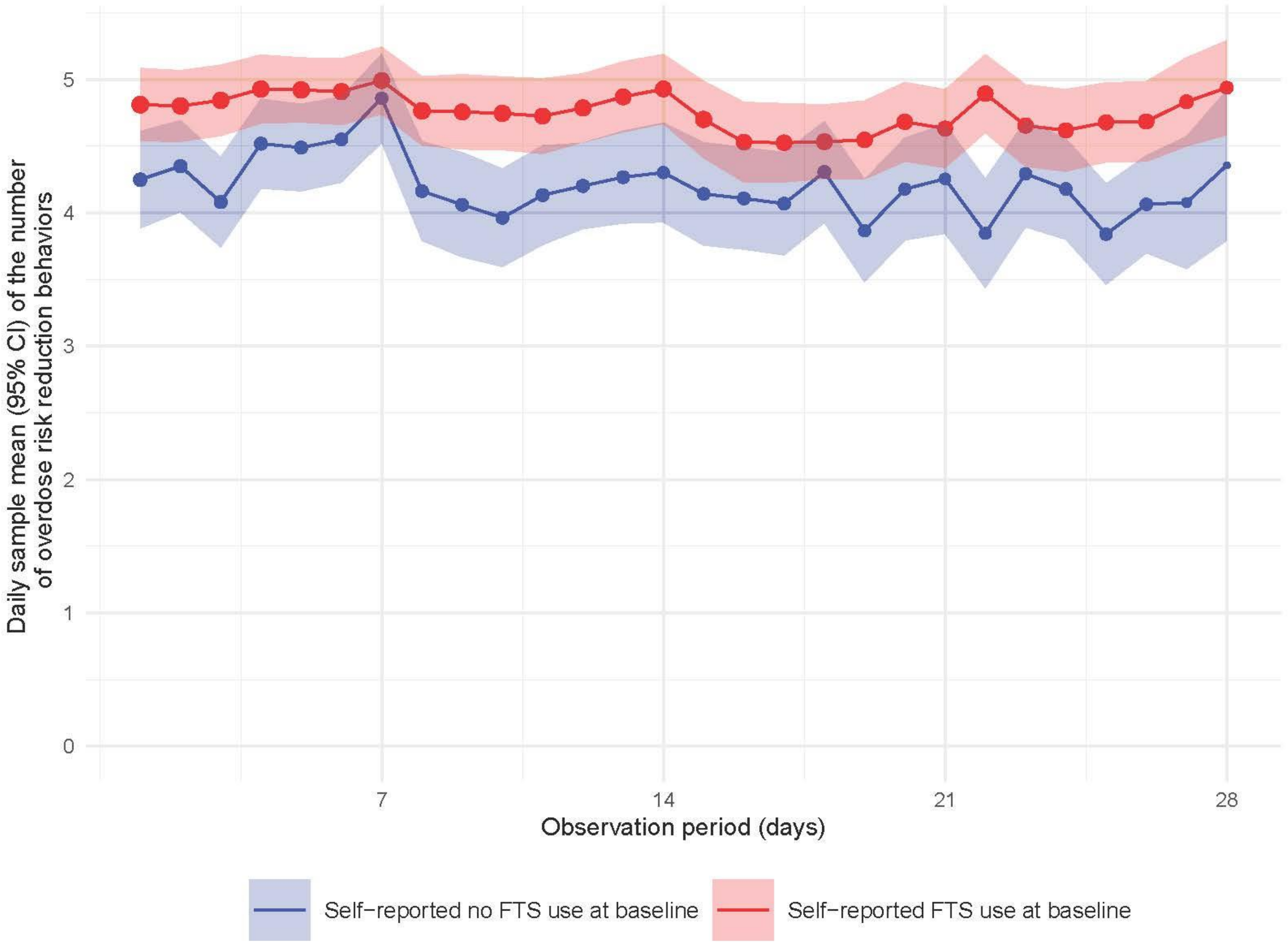

© 2025 Vickers-Smith RA et al. *JAMA Network Open*.

eFigure 3: Daily sample percentage of participants self-reporting each overdose risk reduction behavior during the 28-day observation period by self-reported baseline FTS use.

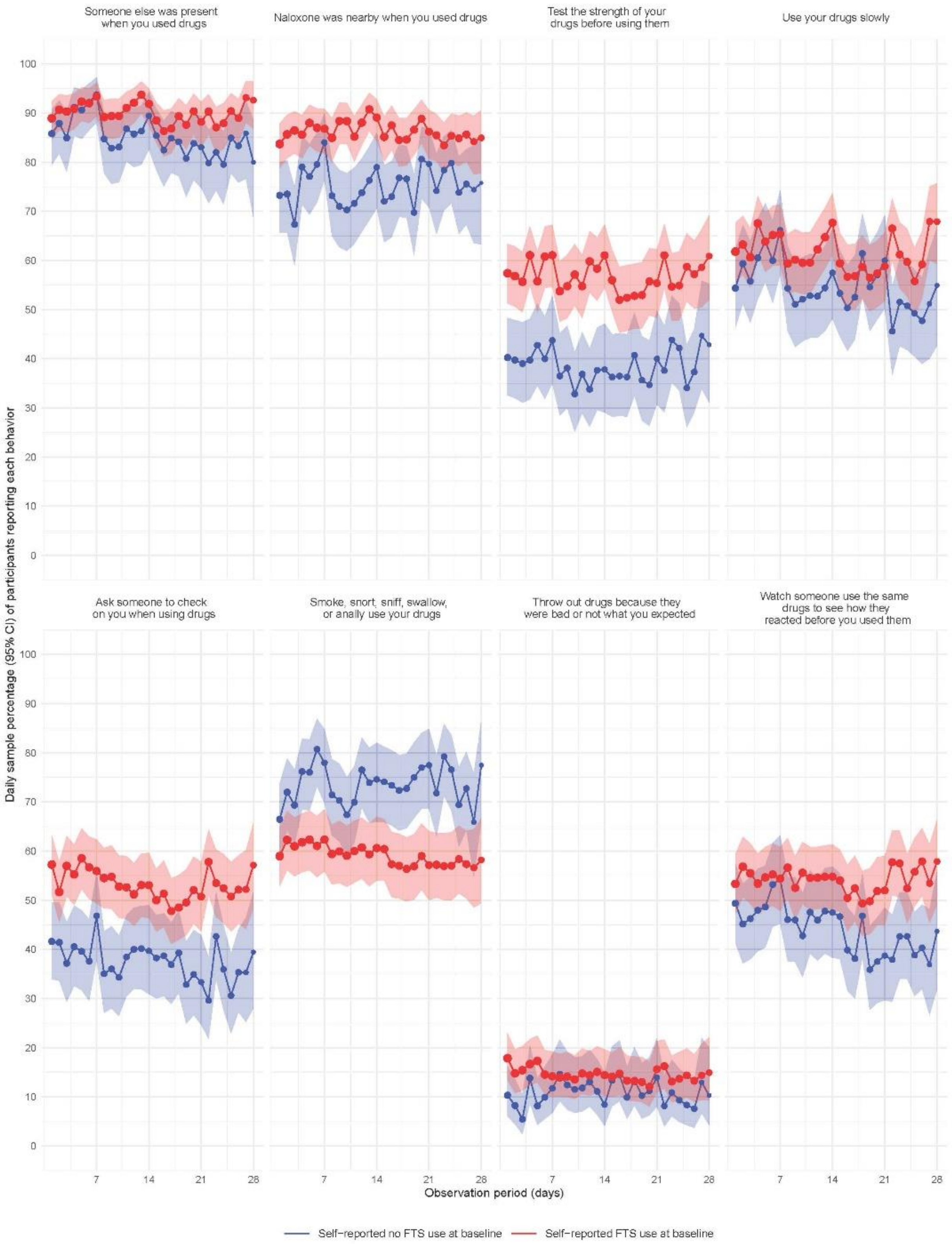

© 2025 Vickers-Smith RA et al. *JAMA Network Open*.

eFigure 4: Daily sample percentage of participants who self-reported a nonfatal overdose during the 28-day observation period by self-reported baseline FTS use.

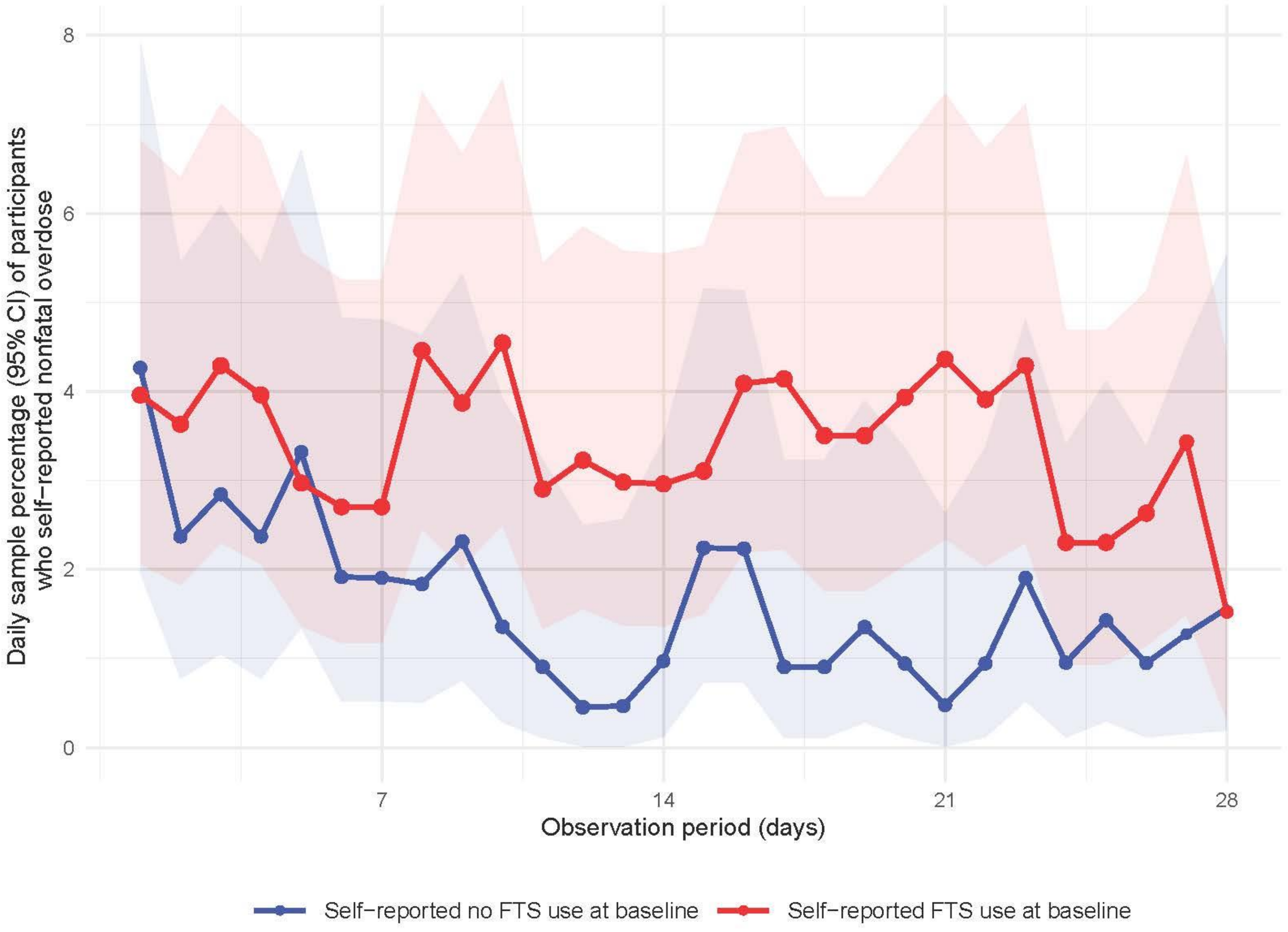

© 2025 Vickers-Smith RA et al. *JAMA Network Open*.

eFigure 5: Modification of the association between self-reported baseline FTS use and self-reported nonfatal overdose by age across the 28-day observation period.

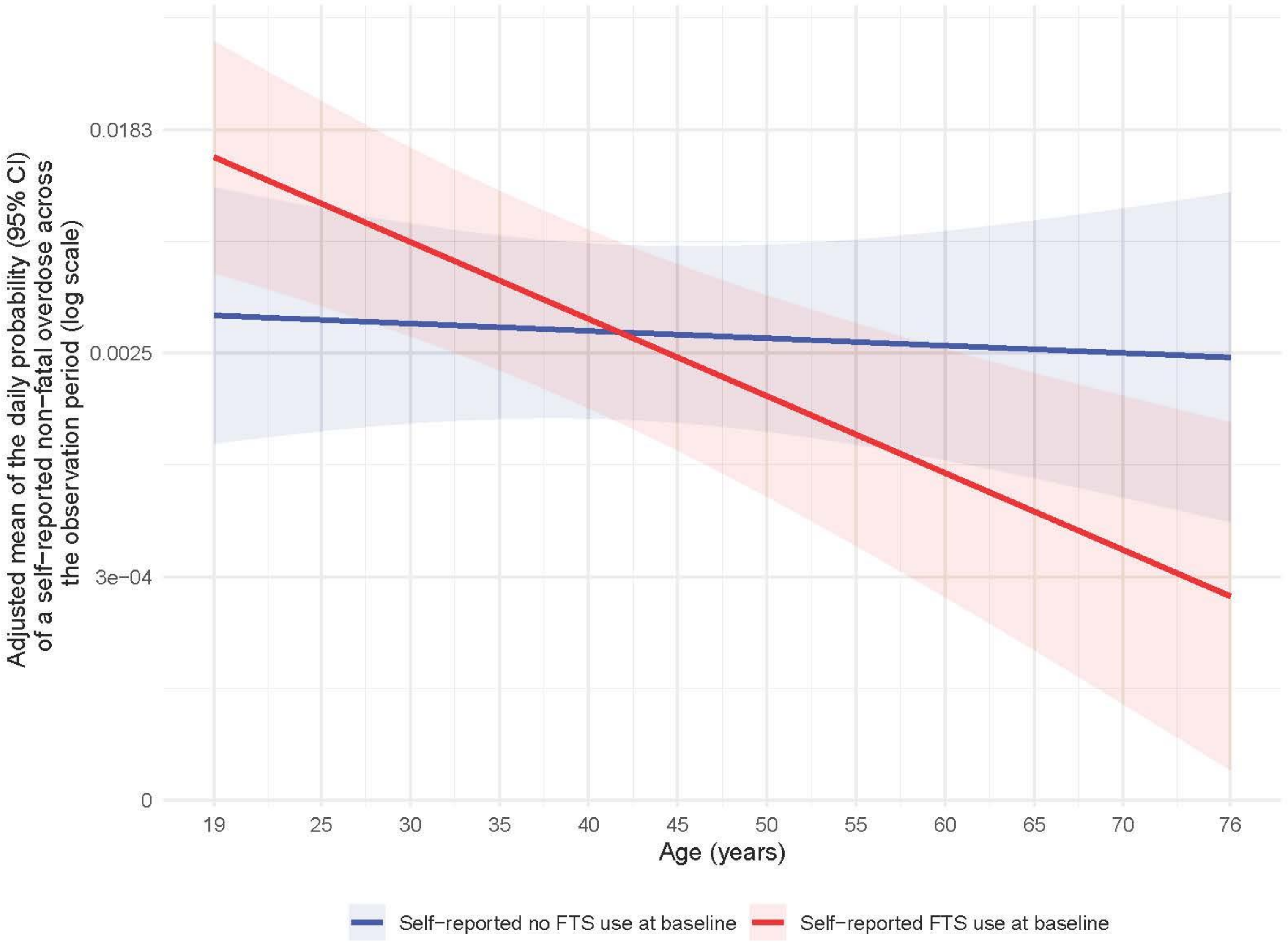

© 2025 Vickers-Smith RA et al. *JAMA Network Open*.
