# Supplementary material for: Fentanyl Test Strip Use and Overdose Risk Reduction Behaviors Among People Who Use Drugs
Source: JAMA Netw Open. 2025 May 13;8(5):e2510077. doi: 10.1001/jamanetworkopen.2025.10077 (PMC12076174; doi:10.1001/jamanetworkopen.2025.10077)
Supplement: Supplement 2. — Data Sharing Statement [file jamanetwopen-e2510077-s002.pdf]

Data Sharing Statement

Vickers-Smith. Fentanyl Test Strip Use and Overdose Risk Reduction Behaviors Among People Who Use Drugs. *JAMA Netw Open*. Published May 13, 2025.
doi:10.1001/jamanetworkopen.2025.10077

Data
Data available: Yes
Data types: Deidentified participant data
How to access data: Data from Kentucky and Ohio will be available. Contact designated representative from each site (Kentucky: Vickers-Smith, rachel.vickers@uky.edu; Ohio: ASC-hcschrr@osu.edu) for further information.
When available: October 31, 2025.

Supporting Documents
Document types: Data dictionaries, data specifications for derived variables, SAS formats, protocol, surveys, statistical analysis plan.

Additional Information
Who can access the data: Due to the sensitive nature of the data collected in this vulnerable population, data and documentation will not be publicly available to ensure the privacy of participants is maintained. Data and documentation may be available upon request from each site representative (Kentucky: Vickers-Smith, rachel.vickers@uky.edu; Ohio: ASC-hcschrr@osu.edu).
